# The Chinese-version of the CARE Measure reliably differentiates between doctors in primary care: a cross-sectional study in Hong Kong

**DOI:** 10.1186/1471-2296-12-43

**Published:** 2011-06-01

**Authors:** Stewart W Mercer, Colman SC Fung, Frank WK Chan, Fiona YY Wong, Samuel YS Wong, Douglas Murphy

**Affiliations:** 1 Primary Care Research, General Practice and Primary Care, University of Glasgow, Glasgow, Scotland, UK; 2 School of Public Health and Primary Care, The Chinese University of Hong Kong, Prince of Wales Hospital, Shatin, N.T., Hong Kong; 3 Quality, Safety and Informatics Research Group, Division of Clinical and Population Sciences and Education, University of Dundee, Dundee, Scotland, UK

**Keywords:** CARE Measure, reliability, consultations, empathy, Hong Kong China, primary care

## Abstract

**Background:**

The Consultation and Relational Empathy (CARE) Measure is a widely used patient-rated experience measure which has recently been translated into Chinese and has undergone preliminary qualitative and quantitative validation. The objective of this study was to determine the reliability of the Chinese-version of the CARE Measure in reliably differentiating between doctors in a primary care setting in Hong Kong

**Methods:**

Data were collected from 984 primary care patients attending 20 doctors with differing levels of training in family medicine in 5 public clinics in Hong Kong. The acceptability of the Chinese-CARE measure to patients was assessed. The reliability of the measure in discriminating effectively between doctors was analysed by Generalisability-theory (G-Theory)

**Results:**

The items in the Chinese-CARE measure were regarded as important by patients and there were few 'not applicable' responses. The measure showed high internal reliability (coefficient 0.95) and effectively differentiated between doctors with only 15-20 patient ratings per doctor (inter-rater reliability > 0.8). Doctors' mean CARE measure scores varied widely, ranging from 24.1 to 45.9 (maximum possible score 50) with a mean of 34.6. CARE Measure scores were positively correlated with level of training in family medicine (Spearman's rho 0.493, p < 0.05).

**Conclusion:**

These data demonstrate the acceptability, feasibility and reliability of using the Chinese-CARE Measure in primary care in Hong Kong to differentiate between doctors interpersonal competencies. Training in family medicine appears to enhance these key interpersonal skills.

## Background

High quality healthcare depends on both technical and interpersonal effectiveness [1-3]. The World Health Organization recently launched a campaign for 'people and patient-centred care [4], representing an important shift in policy direction especially within the Asia pacific region. However, a key practical issue is how best to define and measure patient-centred care. A recent systematic review found a large range of measures that at least partially capture this [5]. Empathy is considered a basic component of the therapeutic relationships and as such is central to patient-centred approaches [6,7]. Empathy is known to enhance a number of patient outcomes, and theory-based modeling suggests both direct and indirect effects [6]. Empathy is thus an important determinant of quality of care [6-9], is influenced by contextual factors such as continuity of care and the available time in the clinical encounter [9-11], and varies significantly between individual clinicians [9-11].

The Consultation and Relational Empathy (CARE) Measure is a patient-rated experience measure (PREM) developed in the United Kingdom [7-9] which has been extensively validated [7-13] and shown to be highly reliable in differentiating between doctors [9-11].

Recent qualitative work explored patients' views on 'good consultations' in primary care in Hong Kong [14] and the key themes mapped closely on to the items in the CARE Measure. We have subsequently carried out extensive work on the translation of the CARE measure into Chinese, and presented preliminary evidence of reliability and validity on 253 primary care patients in Hong Kong [15].

The primary aim of the current study was to determine the reliability of the Chinese version of the CARE Measure in terms of effectively discriminating between doctors by using G-theory analysis [16,17]. A secondary aim was to confirm our recent preliminary findings on reliability and validity on a larger sample of patients [15].

## Methods

A cross-sectional study using a questionnaire which included the Chinese CARE Measure was conducted between July 2008 and February 2009 in primary care clinics in the public health care system run by the Hospital Authority (HA) in Hong Kong. Training in family medicine in Hong Kong is not compulsory, but those who embark on training receive two years of basic training in hospital clinics followed by two years of training in community clinics (basic trainee). They can then sit the fellowship exam of the Hong Kong College of Family Medicine (fellows). Those who pass the fellowship exam then proceed to two further years of higher training to receive the title of specialist in family medicine.

### Setting

Twenty primary care doctors agreed to take part from 5 different General Out Patient Clinics (GOPCs) of the New Territories East Cluster (NTEC), which is a geographical region of health facilities of the Hospital Authority serving 1 million people. The senior doctors and nurses in charge of the clinics agreed to the study. Consent was also obtained from each doctor whose patients were to be recruited into the study. Confidentiality was assured to the doctors (their names were not recorded or known to the research staff, instead each doctor was given a number).

The 20 doctors were a mixture of non-trainees (4), basic trainees (5), fellows (10) and specialists (1) in family medicine, with a range of years of experience (3 to more than 30 years). Six of the twenty doctors were female. Eleven of the doctors were based in a single clinic close to the main teaching hospital in the cluster. The other 9 doctors were distributed evenly across the remaining 4 clinics (2-3 per clinic). Twenty student helpers (mostly medical students) were used to assist in the recruitment of patients and completion of questionnaires.

Consecutive patients (aged 18 or over) were approached in the clinics immediately after the consultation by the student helpers and invited to take part.. Written and verbal information was given to each patient including that the questionnaire was anonymous, responses would be treated in strictest confidence and that no information that they gave would be seen by any of the doctors or other clinic staff. The questionnaire was self-completed whenever possible but if necessary, the student helpers could provide assistance when required and this was recorded on the questionnaire. Patients who did not speak Cantonese as their first language were read the questions by the helpers in Mandarin or English depending on which language the patient spoke.

The completed anonymous questionnaire was then placed in a sealed envelope by the patient after completion and put into a sealed 'ballot box'.

### Patient Questionnaire

In addition to the Chinese CARE measure, the patient questionnaire also collected information on the reason for the encounter ('new problem', 'long-standing problem' or 'both new and old problems'), type of problem discussed (physical, psychological, social, administrative), how many problems were discussed, if the patient was seen by their usual doctor, how well the patient knew the doctor and approximately how long the consultation lasted. Self-assessed general health over the previous 12 months, and any long-term illness, health problem or disability, was recorded. Number and type of chronic diseases were also recorded. All these variables have previously been used in research into the CARE Measure [9,18] including our recent work on the Chinese CARE measure [15].

After the 10 CARE Measure items, the questionnaire asked 'For the problem(s) you were seeing the doctor about today, are the doctors' attitudes and skills listed above [in the CARE Measure] important to you?' Respondents were invited to tick one of four responses--'not important', 'of minor importance', 'moderately important' and 'very important'. The questionnaire then listed the 10 CARE Measure items again and asked respondents to indicate how relevant each item was to them when consulting a primary care doctor, with response options of 'yes', 'no' and 'neutral'. Again these questions have been used successfully before in our pilot work on the Chinese CARE Measure [15].

Data were obtained from the Hospital Authority on the age and gender distribution of all patients attending GOPC clinics over a 12-month period (April 2008-April 2009) so a comparison could be made with the characteristics of the patients who actually participated in the study.

### Ethical issue

Ethical approval was obtained from the NTEC ethics committee of the HA.

### Data Analysis

Descriptive analysis was performed on patient and consultation characteristics. The relevance of the CARE Measure to patients was assessed from their views on its importance overall and importance of each item and by the number of missing and 'not applicable' scores recorded [9-11].

The ten questions in the CARE Measure are rated on a 5-item response scale from 'poor' to 'excellent' by patients in response to the question 'How was the doctor at?' (e.g. item 1: making you feel at ease') with a score of 1 for 'poor' and 5 for 'excellent'. The total score is then calculated by adding up the ten item scores (and can range from 10 to 50). If responses contained missing values or 'not applicable' we re-calculated total score by calculating the average item score and multiplying by 10. Although there are many ways to deal with missing data [19] we have previously shown that this method of dealing with missing or 'not applicable' responses gives similar total scores compared with other approaches such as excluding questionnaires with any missing or 'not applicable' and has the advantage of maximizing sample size [9-12].

Psychometric properties of the CARE measure were examined to confirm earlier findings [15] on a larger sample. The key analysis was the reliability of the measure both in terms of internal reliability and inter-rater reliability (the number of questionnaires required per doctor to attain a reliable score on each doctor) so that the ability of the measure to effectively discriminate between doctors could be ascertained. The reliability (overall, inter-patient and internal consistency) of CARE was assessed using generalisability theory (using urGENOVA software) [16,17]. In each case, doctor was the facet of differentiation (i.e. object of measurement). Raters (patients) were nested within physician. All formulae and variance components are available from the authors upon request. Decision (D) studies were conducted to determine the number of observations required to achieve a reliability of 0^.^8 [17].

The remainder of the statistical analysis was carried out using SPSS software. Differences between groups were analysed by using appropriate parametric and non-parametric tests, and correlations were measured with Spearmann's rho. The latter was chosen in preference to Pearson's correlations as many of the variables included in the correlations were non-parametric. Muli-linear regression analysis was performed using the stepwise approach.

## Results

### Patient characteristics

984 patients took part in the study, with an average response rate of 84% (range 57% to 98% per doctor). The number of patients participating per doctor ranged from 45 to 51. Table 1 shows the characteristics of the participating patients. The gender and age group distribution of the participating patients (table 1) was very similar to that of all patients attending GOPC clinics in the New Territories East Cluster in the previous year (out of 420,295 patients aged 18 years or above who had attended in the previous year, 57% were female, 13% were aged 18-44 years, 49% were aged 45 - 65 years, and 38% were aged above 65 years). Participating patients were mainly married, with limited educational levels and on low to medium incomes (table 1). In terms of ethnicity, 955 out of the 984 (97.3%) spoke Cantonese as their first language (1% spoke Mandarin and 1.7% 'other').

**Table 1:** Demographic data of participating patients

	n (%)
**Age group**	
18-44 years	135 (13.8%)
45-65 years	462 (47.2%)
> 65 years	381 (39.0%)
**Gender**	
Male	426 (43.3%)
Female	552 (56.1%)
**Marital status**	
Single	83 (8.4%)
Married/Cohabitant	768 (78.0%)
Separated	5 (0.5%)
Divorced	26 (2.6%)
Widowed	78 (7.9%)
**Education level**	
No education	146 (14.8%)
Primary school level	344 (35.0%)
Secondary school level	410 (42.7%)
Tertiary education	60 (6.1%)
Others	8 (0.8%)
**Monthly household income (HKD)**	
On CSSA (welfare)	47 (4.8%)
≤ $5000	136 (13.8%)
$5000-10000	152 (15.4%)
$10001-20000	190 (19.3%)
$20001-30000	117 (11.9%)
$30001-40000	47 (4.8%)
$40001-50000	15 (1.5%)
$50001-60000	7 (0.7%)
≥ $60001	8 (0.8%)
Not sure	254 (25.8%)

Table 2 shows the chronic disease profiles of attending patients. Over 80% of patients reported a chronic disease, most commonly hypertension, diabetes, and high cholesterol. More than half of those with a chronic disease had multimorbidity (2 or more conditions). The majority of patients assessed their general health over the last 12 months as fair.

**Table 2:** Patients disease profiles and self-reported health status

	n (%)
**Disease**	
Hypertension	535 (54.3%)
Diabetes	228 (23.2%)
High cholesterol	104 (10.6%)
Angina/heart attach	38 (3.9%)
Stroke/mini-stroke	27 (2.7%)
Heart failure	9 (0.9%)
Chronic bronchitis/Asthma	46 (4.6%)
Kidney disease	36 (3.7%)
Back problems	40 (4.1%)
Arthritis	93 (9.5%)
Liver disease	14 (1.4%)
Cancer	13 (1.3%)
Eczema/psoriasis	44 (4.5%)
Anxiety/depression	16 (1.6%)
Irritable bowel syndrome	6 (0.6%)
Migraine	1.3 (1.3%)
Others	144 (14.4%)
**Multimorbidity**	
No chronic diseases	163 (16.6%)
1 chronic disease	400 (40.7%)
2 chronic diseases	286 (29.0%)
> 2 chronic diseases	135 (13.7%)
**Limits daily activities**	
Yes	315 (32.5%)
**General Health over last 12 months**	
Very Bad/Bad	142 (14.5%)
Fair	523 (53.2%)
Good/Very Good	318 (32.3%)

### Consultation characteristics

Table 3 shows the consultation characteristics of the participating patients. The reason for attendance was mainly for physical problems with few attending for psychological or other problems. Three out of four patients were attending for treatment of chronic conditions (with or without a new problem). Most patients (56%) only discussed one problem in the consultation (mean 1.57 (SD 0.71)/median 1.0), consultations lengths averaged 5.5 (SD2.94)/median 5.0 minutes and continuity of care (on a five point scale from 1 to 5) was rare and only 10% of patients felt they knew the doctor quite well (4) or very well (5) with a mean of 1.7 (SD1.09)/median 1.0.

**Table 3:** Consultation characteristics

	n (%)
**Reason for consultation**	
Physical problem	973 (98.9%)
Psychological problem	11 (1.1%)
Social problem	2 (0.2%)
Administrative issue	14 (1.4%)
Others	6 (0.6%)
**Number of problems discussed**	
One	550 (55.9%)
Two	301 (30.6%)
Three or more	128 (13.0%)
Mean (SD)/Median	
**Nature of the problem**	
New (acute) illness	241 (24.5%)
Old (chronic) illness	640 (65.0%)
Both old and new	98 (10.0%)
**Duration of consultation**	
< 3 minutes	72 (7.4%)
3-5 minutes	638 (65.4%)
6-8 minutes	102 (10.5%)
9-10 minutes	136 (13.9%)
11-15 minutes	23 (2.4%)
> 15 minutes	5 (0.5%)
Mean (SD)/Median	
**Continuity of care**	
Yes, usual doctor	237 (24.1%)
Not the usual doctor	450 (45.7%)
No usual doctor	290 (29.5%)
*Knows the doctor:*	
Not at all well	649 (66.5%)
Not well	144 (14.8%)
Neutral	86 (8.8%)
Quite well/Very well	97 (10.0%)
Mean (SD)/Median	

### Patients' views on the importance of the CARE Measur*e*

The importance of the CARE Measure overall to patients was recorded in 979 patients out of the 984 (5 missing values). 924 out of 979 (94.4%) of the patients felt that the attitudes and skills of doctors as described in the CARE Measure were important to their current consultation (57.6% responded 'of moderate importance', and 36.6% responded 'very important'. within the CARE Measure were important). Less than 1% of patients felt that the attitudes and skills were 'not important'.

Patients' views on the importance of each individual CARE Measure item to their current consultation are shown in table 4. The percentage of patients recording 'important' ranged from 83.7% to 98.0%.

**Table 4:** Patients' views on the importance of individual CARE Measure items

CARE Measure item:	Patients' views on the importance of CARE Measure items in the current consultation n (%)		
	Important	Not important	Not sure
Item 1 **Making you feel at ease ......**	938 (96.2%)	17 (1.7%)	20 (2.1%)
Item 2 **Letting you tell your "story" ......**	918 (94.0%)	32 (3.3%)	27 (2.8%)
Item 3 **Really listening ......**	958 (98.0%)	11 (1.1%)	9 (0.9%)
Item 4 **Being interested in you as a whole person ......**	816 (83.7%)	72 (7.4%)	87 (8.9%)
Item 5 **Fully understanding your concerns ......**	897 (91.8%)	36 (3.7%)	44 (4.5%)
Item 6 **Showing care and compassion ......**	929 (95.2%)	20 (2.0%)	27 (2.8%)
Item 7 **Being Positive ......**	943 (96.7%)	13 (1.3%)	19 (1.9%)
Item 8 **Explaining things clearly**	945 (97.1%)	14 (1.4%)	14 (1.4%)
Item 9 **Helping you to take control ......**	886 (91.1%)	31 (3.2%)	56 (5.8%)
Item 10 **Making a plan of action with you ......**	815 (83.7%)	56 (5.7%)	103 (10.6%)

**Figure 1:**
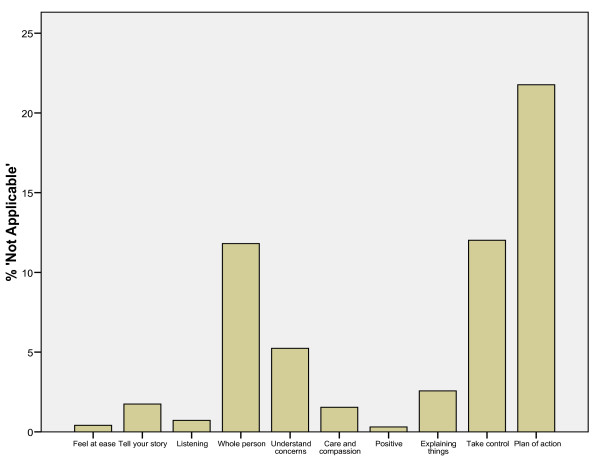
**Percentage of 'not applicable' response to CARE measure items**.

The proportions of patients with "not applicable" responses to the CARE Measure items ranged from 0.3% for item 7 ("being positive") to 21.5% for item 10 "Making a plan of action with you" with an average of 5.7% across all ten items (see figure 1). The number of missing values ranged from 0.1% to 0.5% with an average of 0.2% (results not shown).

### CARE Measure scores

The mean CARE measure score across the 984 patients was 34.6 (SD 8.75) with little skew or kurtosis (skew -0.51; kurtosis -0.55) and a median of 35.0. Scores ranged from 10 (minimum possible score) to 50 (maximum possible score).

The distribution of responses across each item within the CARE measure was reasonably normal, with an average of 18.5% of patients recording 'poor' or 'fair' responses (ranging from 12.3% for item 7 "Being positive" to 28.2% for item 4 "Being interested in you as a whole person") up to an average of 16.1% recording 'excellent' (ranging from 10.4% for item 10 "making a plan of action" to 21.6% for item 8 "Explaining things clearly"). Thus there was no evidence of any ceiling effects (full results not shown).

### Factors influencing CARE Measure scores

We examined CARE Measure scores according to the different patient characteristics shown in table 1. Age had a very weak positive correlation with CARE Measure scores (Spearman's rho 0.104, p = 0.001) whereas gender, marital status, educational level, and family income had no significant associations with CARE Measure scores (results not shown).

We also examined CARE Measure scores according to patients' disease and health characteristics (as shown in table 2). Because the number of patients with single diseases was limited and many single diseases had small sample sizes, we did not explore this on a disease by disease basis. However, patients with one or more chronic diseases (of any type) had higher CARE Measure scores than those with no chronic diseases; mean 34.8 (SD 8.7), versus 33.3 (SD 9.0) respectively, p = 0.049. Multimorbidity (number of chronic diseases within an individual) had no effect on CARE Measure score, but self-reported health over the last 12 months was significantly but weakly correlated with CARE measure scores, with those reporting poorer health having lower CARE measure scores (Spearman's rho 0.155, p < 0.001).

In terms of consultation characteristics (table 3) and CARE scores, we found significant but weak associations between CARE score and self-reported consultation length (Spearman's rho 0.128, p < 0.001), knowing the doctor (Spearman's rho 0.103, p < 0.001), and the number of problems the patient discussed (Spearman's rho 0.073, p < 0.05). The nature of the problem also had a significant effect on CARE score, with patients consulting with a new problem having a lower score than those consulting about an old problem; 32.7 (SD 8.3) versus 35.1 (SD 8.8), respectively, p < 0.001.

Because many of the variables found to be significantly associated with CARE measure scores were themselves significantly inter-related (for example age and self-reported health), we performed step-wise multi-linear regression analysis to identify the factors that independently predicted CARE scores, and the relative contribution of these to the variation in scores. Age, self-reported health, knowing the doctor, number of problems discussed, and self-reported consultation length were entered into the model as continuous variables, and chronic disease (or not) and nature of problem (new or old/new and old) were entered as binary variables. Four variables emerged as independent predictors of CARE Measure score (table 5), with an overall model r-squared of 0.083 and adjusted r-squared of 0.079. These four factors were general health, knowing the doctor, self-reported consultation length, and whether consulting for a new or old problem.

**Table 5:** Multiple regression analysis of factors associated with CARE Measure scores

Variable	Effect estimate (un-standardised beta)	95% Confidence intervals	Significance level	R square change
General Health	2.311	1.577 to 3.046	P < 0.001	0.027
Knowing the doctor	1.104	0.605 to 1.603	P < 0.001	0.029
Consultation length	0.338	0.154 to 0.522	P < 0.001	0.015
Acute or chronic problem	2.208	0.957 to 3.460	P < 0.01	0.012

Step-wise regression was performed with CARE measure score as the dependant variable and the following independent variables; age, self-reported general health over the last 12 months, knowing the doctor, number of problems discussed, and consultation length were entered into the model as continuous variables, and chronic disease (or not) and nature of problem (new or old/new and old) were entered as binary variables.

### Reliability of CARE measure: G-Theory analysis

A key aim of the present study was to determine the effectiveness of the Chinese-CARE Measure in discriminating between doctors, and the results of the G-Theory analysis on this are shown in table 6. As expected, the reliability of the measure increased with the number of raters (patients) per doctor. The results indicate that the measure overall was able to discriminate effectively between the doctors with a feasible number of patient ratings per doctor. For a given number of patients per doctor, the reliability co-efficient was slightly higher for the non-family doctor group (non-trainees and basic trainees) compared with the family doctor group (fellows and specialist in family medicine) but even for the latter a reliability of 0.8 was achieved with less than 30 patients per doctor.

**Table 6:** Reliability of the Chinese CARE Measure in differentiating between doctors (G-Theory)

Number of patients per doctor	Reliability (all doctors)	Reliability (non-family doctors)	Reliability (family doctors)
1	0.18	0.38	0.16
10	0.77	0.84	0.64
15	0.82	0.88	0.71
20	0.85	0.90	0.76
30	0.89	0.92	0.81
40	0.90	0.94	0.84

The internal reliability (Cronbach's alpha) of the measure was also high at 0.95 in both groups.

**Figure 2:**
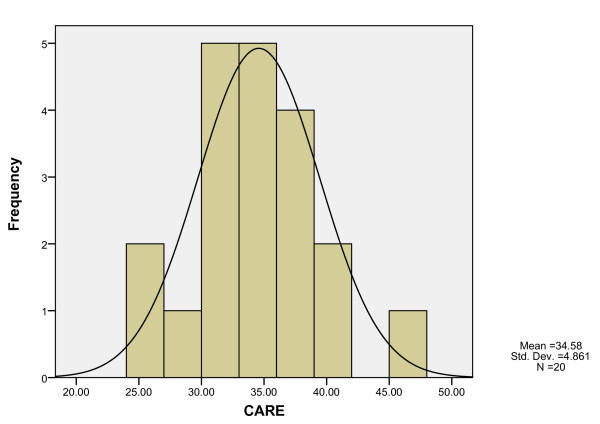
**Distribution of doctors' mean CARE measure scores**.

Doctors mean CARE measure scores ranged from 24.1 to 45.9 and were normally distributed (Figure 2). Mean CARE measure score was correlated significantly with grade (Spearman's rho 0.493, p < 0.05), with non-trainees having the lowest scores, basic trainees in family medicine having intermediate scores, and fellows of family medicine having the highest; mean CARE scores 29.9 (SD 3.99), 33.9 (SD 4.61) and 36.8 (SD 4.46), respectively, p = 0.055). Gender, years working as a medical officer, and clinic setting were not significantly related to doctors mean CARE scores (results not shown).

## Discussion

Empirical research and theoretical analysis has shown clinical empathy to be an important determinant of quality of care [6,7] which varies significantly between physicians [9-11]. The Chinese-version of the CARE Measure has been previously shown to capture patients' views on physician empathy [14] in a valid and reliable way [15]. The primary aim of the present study was to determine the reliability of the Chinese-version of the CARE Measure in differentiating between doctors in a primary care setting. We achieved this by collecting data on almost one thousand patients attending twenty doctors with differing levels of training in family medicine in 5 public clinics in Hong Kong. Given the high response rate (84%) and the close agreement between the age and gender distribution of participating patients compared with all patients attending the clinics over the previous year, it seems likely that this was a highly representative patient sample. The patients attending the public primary care clinics were generally middle-aged to elderly, most had one or more chronic physical diseases, and were mainly consulting about these conditions. These patient characteristics also generally agree with our recent findings from a smaller sample in the same setting [15].

Patients viewed the attitudes and skills reflected in the Chinese-CARE measure items as being highly relevant to their current consultation, and rated each item highly in terms of importance. These ratings of importance are similar to, but slightly higher than, our previous findings on a smaller sample in the same setting [15]. The relevance of the items in the measure was also reflected by the low number of missing values and 'not applicable' responses overall. However, again in line with our previous findings [15] there was some variation between items, with items 4 (whole-person approach) and 10 (shared plan of action) having the highest percentages of 'not applicable' responses and the lowest ratings of importance. This may relate to low expectations of both holistic care and involvement in decisions by patients in this setting in Hong Kong [14,15]. Further work is required on the Chinese-CARE measure in other primary care settings (such as the private general practice and family medicine setting, and the traditional Chinese Medicine system) to see if the pattern is the same or different with the public system.

The reliability of the Chinese-CARE Measure as determined by G-Theory analysis showed high internal reliability, and high inter-rater reliability, indicating that the measure does indeed effectively and reliably differentiate between doctors. For doctors trained in family medicine a reliability of over 0.80 was achieved with ratings from 30 patients per doctor. For non-family medicine trained doctors the sample size required per doctor was even less. This makes the Chinese-CARE Measure highly feasible as a tool to measure performance at doctor level, given that the collection of data from 30 patients is not an onerous task.

### Relevance to literature

The reliability of the original (English version) of the CARE measure has also been demonstrated using G-Theory in both primary and secondary care settings [9-11]. In these UK studies, 40-50 patients were required per doctor to attain a reliable CARE Measure score whereas in the present study highly reliable scores were attained in the family doctor group with somewhat fewer (around 30) patients per doctor. As indicated in the results, the level of training in family medicine correlated positively and significantly with mean CARE Measure scores at doctor level. In our previous work in the UK, our reliability studies on the CARE Measure have only compared doctors of the same grade, i.e., fully qualified general practitioners [9], GP registrars [20], or consultant specialists [10,11].

The high relevance of the Chinese-CARE measure to patients supports our previous research in Hong Kong [14,15]. Similar findings have been reported for the original CARE measure in the UK [8,9]. The factors associated with the Chinese-CARE Measure scores also accords with our previous smaller study in Hong Kong, in that a weak but statistically significant positive effect of self-reported consultation length and continuity (knowing the doctor well) on Chinese-CARE measure scores were demonstrated in both studies [15]. However, in the present study, multi-regression analysis also showed an association with general health and whether the patient consulted for an acute or chronic problem. The associations with time (whether reported by the patient or measured by the doctor) and continuity have been demonstrated previously in UK studies [9-11], whereas effects of general health and nature of problem (acute or chronic) have not been found [9]. Further work is required to explore the reason behind these associations in the Hong Kong setting. However, it is important to note that the explanatory power of the model was low in the present study, with all four factors combined (time, continuity, general health, acute or chronic problem) explaining less than 9% of the variation in Chinese-CARE measure scores. Thus case-mix issues are likely to be relatively unimportant when comparing scores across different doctors using the Chinese-CARE Measure.

### Strengths and weaknesses

An important strength of the present study was that we attained high response rates amongst patients and participants were representative of patients attending the GOPC clinics. We were also able to collect almost 50 Chinese-CARE Measure scores for all participating doctors. The number of doctors who took part was sufficient to detect major differences in CARE Measure scores between doctors with a high degree of reliability.

An additional strength is that this study builds on previous studies on the relevance of the measure to Chinese patients [14,15], and further supports the reliability and validity of the Chinese-CARE Measure.

Limitations of the study include the fact that patients were recruited on a consecutive basis rather than randomly, although as we have shown, their characteristics were similar to the total population of patients attending the clinics in the preceding year. Also only 20 doctors of differing levels of training in family medicine took part, so whether the differences found between those with and without family medicine training are generalisable cannot be established and further work is required on a large, representative sample of doctors to explore this finding further. Although, the gradient in mean Chinese-CARE Measure scores per doctor associated with level of training suggests the value of training in family medicine in this primary care setting, future work is required to establish this on a larger sample and with more advanced statistical methods such as multi-level modelling to account for potential cluster effects (which was beyond the scope of the present study). Given that most patients have long-term conditions, this finding could have considerable policy relevance at a time when the Hong Kong Government is actively promoting the primary care management of long-term conditions, based around a family doctor model.

## Conclusions

The reliability of the Chinese-version of the CARE Measure in differentiating between doctors in a primary care setting in Hong Kong was assessed. The measure effectively differentiates between doctors with a feasible number of patient ratings per doctor. Doctors' mean CARE Measure scores were positively correlated with level of training in family medicine. We conclude that the Chinese-CARE Measure is an acceptable, feasible tool to differentiate between doctors interpersonal competencies.

## Competing interests

The authors declare that they have no competing interests.

## Authors' contributions

All authors participated in concept and designing the study and data interpretation but SWM took the lead role in this. SWM and CSCF drafted the manuscript. DM performed the reliability analysis. All authors read and approved the final manuscript.

## Pre-publication history

The pre-publication history for this paper can be accessed here:

http://www.biomedcentral.com/1471-2296/12/43/prepub

## References

[B1] Campbell SM Rowlands MO Buetow S Defining quality of care Soc Sci Med 2000 51 1611 1625 10.1016/S0277-9536(00)00057-5 11072882

[B2] Howie JGR Hemey DJ Maxwell M Quality, core values and general practice consultation: issues of definition, measurement and delivery Fam Pract 2004 21 458 468 10.1093/fampra/cmh419 15249538

[B3] Wensing M Jung HP Mainz J Olesen F Grol R A systematic review of the literature on patient priorities for general practice care. Part 1: description of the research domain Soc Sci Med 1998 47 1573 1588 10.1016/S0277-9536(98)00222-6 9823053

[B4] International symposium on people-centred health care: reorienting health systems in the *21*^*st *^*century- Tokyo international forum* 2007 Tokyo 25 November 2007

[B5] Hudon C Fortin M Haggerty JL Lambert M Poitras M Measuring patients' perceptions of patient-centred care: a systematic review of tools for family medicine Ann Fam Med 2011 9 155 164 10.1370/afm.1226 21403143 PMC3056864

[B6] Neumann M Bensing J Mercer S Ernstmann N Ommen O Pfaff H Analyzing the "nature" and "specific effectiveness" of clinical empathy A theoretical overview and contribution towards a theory-based research agenda Patient Education and Counseling 2009 74 339 46 10.1016/j.pec.2008.11.013 19124216

[B7] Mercer SW Reynolds W Empathy and quality care Br J Gen Pract 2002 52 Suppl S9 S12 12389763 PMC1316134

[B8] Mercer SW Maxwell M Heaney D Watt GCM The consultation and relational empathy (CARE) measure: development and preliminary validation and reliability of an empathy-based consultation process measure Family Practice 2004 21 699 705 10.1093/fampra/cmh621 15528286

[B9] Mercer SW McConnachie A Maxwell M Heaney D Watt GCM Relevance and practical use of the Consultation and Relational Empathy (CARE) Measure in general practice Family Practice 2005 22 328 334 10.1093/fampra/cmh730 15772120

[B10] Mercer SW Hatch DJ Murray A Murphy DJ Eva HW Capturing patients' views on communication with anaesthetists: the CARE Measure Clinical Governance: An International Journal 2008 13 128 137 10.1108/14777270810867320

[B11] Mercer SW Murphy DJ Validity and reliability of the CARE Measure in secondary care Clinical Governance: An International Journal 2008 13 4 269 283 10.1108/14777270810912969

[B12] Mercer SW Neumann M Wirtz W Fitzpatrick B Vojt G Effect of General Practitioner empathy on GP stress, patient enablement, and patient-reported outcomes in primary care in an area of high socio-economic deprivation in Scotland - A pilot prospective study using structural equation modelling Patient Education and Counseling 2007 73 240 245 10.1016/j.pec.2008.07.022 18752916

[B13] Neumann M Wirtz M Bollschweiler E Mercer SW Warm M Wolf J Pfaff H Determinants and patient-reported long-term outcomes of physician empathy in oncology: a structural equation modeling approach Patient Education & Counseling 2007 69 1-3 63 75 10.1016/j.pec.2007.07.003 17851016

[B14] Fung CSC Mercer SW A qualitative study of patients' views on quality of primary care consultations in Hong Kong and comparison with the UK CARE Measure BMC Fam Med 2009 10 10 10.1186/1471-2296-10-10 PMC2633320 19173724

[B15] Fung CSC Hua A Tam L Mercer SW Reliability and validity of the Chinese version of the CARE Measure in a primary care setting in Hong Kong Family Practice 2009 26 398 406 10.1093/fampra/cmp044 19587027

[B16] Brennan RL Generalizability theory 2001 New York: Springer-Verlag http://www.education.uiowa.edu/casma/computer_programs.htm Accessed on 13 December 2009.

[B17] Streiner D Norman G Health measurement scales: A practical guide to their development and use 2008 4 New York: Oxford University Press

[B18] Mercer SW Watt GCM The inverse care law: Clinical primary care encounters in deprived and affluent areas of Scotland Annals of Family Medicine 2007 5 503 510 10.1370/afm.778 18025487 PMC2094031

[B19] Schafer Jl Graham JW Missing data: our view on the state of the art Psychological methods 2002 7 2 147 177 12090408

[B20] Hox JJ Multilevel analysis. Techniques and applications 2010 2 New York: Routledge

[B21] Murphy DJ Bruce DA Mercer SW Eva KW The reliability of workplace-based assessment in postgraduate medical education and training: a national evaluation in general practice in the United Kingdom Adv in Health Sci Educ 2009 14 219 232 10.1007/s10459-008-9104-8 18306052

